# Comprehensive evaluation of physicochemical properties and antioxidant activity of *B. subtilis*‐fermented polished adlay subjected to different drying methods

**DOI:** 10.1002/fsn3.1508

**Published:** 2020-03-14

**Authors:** Anyan Wen, Likang Qin, Haiying Zeng, Yi Zhu

**Affiliations:** ^1^ College of Life Science Guizhou University Guiyang China; ^2^ School of Liquor and Food Engineering Guizhou University Guiyang China; ^3^ Key Laboratory of Agricultural and Animal Products Storage and Processing of Guizhou Province Guiyang China; ^4^ National and Local Joint Engineering Research Center for the Exploition of Homology Resources of Medicine and Food Guiyang China; ^5^ Plant Protection and Plant Quarantine Station of Guizhou Province Guiyang China

**Keywords:** antioxidant activity, *B. subtilis‐*fermented polished adlay, drying methods, physicochemical properties, principal component analysis

## Abstract

The physicochemical properties and antioxidant activity of *B. subtilis*‐fermented polished adlay (BPA) subjected to different drying methods (hot‐air drying, HAD; infrared‐radiation drying, IRD; vacuum drying, VD; microwave‐vacuum drying, MVD; and freeze‐vacuum drying, FVD) were evaluated in this study. Results showed FVD was ideal for maintaining the natural appearance and higher contents of proximate compositions, free fatty acids, tetramethylpyrazine (6.91 mg/g DW), coixol (0.62 mg/g DW), coixenolide (4.21% DW), coixan (35.10% DW), and triterpenoids (17.41 mg/g DW). The higher contents of total phenolics and flavonoids, stronger antioxidant activity, and higher color differences were observed in HAD and IRD samples. MVD displayed the shorter drying time, higher γ‐aminobutyric acid content, and higher retention ratios of tetramethylpyrazine (75.54%), coixol (87.10%), coixenolide (98.57%), and coixan (99.11%). Pearson's correlation coefficient exhibited that the positive correlation between the contents of phenolics and flavonoids and the antioxidant activities of all dried BPA samples was observed (*R* ^2^ > 0.881, *p* < .05). Principal component analysis showed that the top three categories of comprehensive quality were FVD‐, MVD‐, and VD‐treated BPA samples. In conclusion, MVD should be a potential preservation method to obtain high‐quality dried BPA for short drying time and high comprehensive quality.

## Graphical abstract

The physicochemical properties and antioxidant activity of B. subtilis‐fermented polished adlay (BPA) treated by different drying methods were evaluated in this study. Through analysing comprehensive quality, microwave‐vacuum drying should be a potential preservation method to obtain high‐quality dried BPA for short drying time and high comprehensive quality.

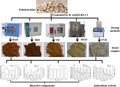

## 1 INTRODUCTION

Adlay (*Coix lachryma‐jobi*), a soft shelled seed crop widely cultivated in China, Japan, Thailand, and Burma, has long been consumed as nourishing food and traditional oriental medicine (Huang et al., 2019). As for the nutrition content, the major component of adlay is starch (~54.26%–58.15%), and the contents of lipid, protein, and essential amino acids for human (lysine and methionine) are higher compared with other cereals (Lin et al., 2009). Meanwhile, a great number of bioactive components had been detected in adlay, including coixol, coixenolide, coixan, γ‐aminobutyric acid, flavonoids, phenolics, triterpenoids, steroids, lignans, and lactams (Ting et al., 2019). Several studies claimed that adlay had various beneficial functions, including antioxidant, anti‐inflammation, anticancer, enhancing immunological activity, regulating endocrine functions, antidiabetes, antiobesity, and modulating gut microbiota (Zhu, 2017). Owing to its multiple nutritional and health benefits, the consumption demand for adlay has continued to increase.

Fermentation by fungi, yeast, and bacteria has been an inexpensive and effective process that improves nourishing and functional food ingredients, and functional properties. *Monascus*‐fermented adlay with high contents of mevinolin, α‐tocopherol, and γ‐oryzanol displayed stronger antioxidant activity and hypolipidemic effects than unfermented adlay (Ding, Pu, & Kan, 2017; Pattanagul, Pinthong, Phianmongkhol, & Tharatha, 2008; Yang, Tseng, Lee, & Mau, 2006). Additionally, Wang, Lin, and Wu (2011, 2014) observed that the changes in antioxidant status, lipid metabolism, and intestinal microflora were significantly improved by *Bacillus*‐fermented adlay. In previous study, we found that adlay was fermented by *B. subtilis*, which resulted in high levels of tetramethylpyrazine, γ‐aminobutyric acid, phenolics, flavonoids, triterpenoids, free amino acids, and fatty acids (Wen et al., 2019). Due to high health‐beneficial components and water content of *B. subtilis‐*fermented adlay, it is far more susceptible to bacterial and fungal contamination. Therefore, the major challenge is that *B. subtilis‐*fermented adlay must be stored using dehydration methods.

Studies have shown that drying process was used to effectively restrain the growth of other microorganisms and minimize plenty of moisture‐mediated degradation reactions. Hot‐air (HAD), infrared‐radiation (IRD), vacuum (VD), microwave‐vacuum (MVD) and freeze‐vacuum (FVD) drying have been commonly used in food processing and preservation. Pham, Nguyen, Vuong, Bowyer, Scalett (2017), Pham, Vuong, Bowyer, and Scarlett (2017) observed that VD maintained the higher contents of proanthocyanidins, flavonoids, and phenolic compounds, and stronger antioxidant activities in *Catharanthus roseus* than HAD and IRD. Sui, Mu, Sun, and Yang (2019) demonstrated that sweet potato leaves treated by FVD showed higher contents of vitamin, mineral, and phenolics, and stronger antioxidant activity than that by MVD and HAD. Thus, the appropriate method for drying *B. subtilis*‐fermented adlay was not directly obtained according to previous studies. And until now, no or few studies were available examined the physicochemical properties and bioactivities of *B. subtilis*‐fermented adlay treated by different drying methods.

In the present study, the nutritional and bioactive (tetramethylpyrazine, acetoin, coixol, coixenolide, coixan, γ‐aminobutyric acid, triterpenoids, phenolics, and flavonoids) components and antioxidant activity of *B. subtilis*‐fermented polished adlay (BPA) dried by five methods were detected for the first time. Furthermore, Pearson's correlation coefficient was used to evaluate the correlation between the bioactive components and antioxidant activity. Additionally, the comprehensive qualities of BPA processed by five drying methods were evaluated by principal component analysis (PCA). The results of this study will present scientific basis for choosing appropriate methods for drying BPA, which will maintain the best possible contents of nutritional and bioactive components, and promote the industrial application of BPA.

## 2 MATERIALS AND METHODS

### 2.1 Materials and chemicals

Polished adlay used in this study was provided by Guizhou Renxin Agricultural Development Co., Ltd., and was sealed in plastic bags and stored at 4°C until use. *B. subtilis* BJ3‐2 was procured from Dr. Wu (College of life sciences, Guizhou University). BPA was prepared as described by Wen et al. (2019). Tetramethylpyrazine, acetoin, γ‐aminobutyric acid, oleanolic acid, gallic acid, rutin, 2,2′‐azinobis(3‐ethylbenzothiazoline‐6‐sulfonic acid) (ABTS), and 2,2‐diphenyl‐1‐picrylhydrazyl (DPPH) were purchased from Beijing Solarbio Science & Technology co., Ltd. (Beijing, China). All other chemicals were of analytical grade.

### 2.2 Drying process

The BPA samples were subjected to HAD, IRD, VD, MVD, and FVD until the final moisture content was approximately 8.50 ± 1.00 g/100 g dry weight. And FVD was set as reference object due to that it maintained dried samples with high nutritional and functional components (Wen et al., 2019). All dried samples were placed onto the plates with 0.5 cm thickness and then followed with drying methods as displayed in Table 1. After drying, BPA samples were blended into powder and screened through a 60 mesh sieve. All dried BPA powdered samples were stored at 4°C until used.

**Table 1:** Maximum final moisture content and time

Drying methods	Temperature (°C)	Vacuum pressure (Mpa)	Time (hr)	Wet weight (g)	Drying weight (g)	Maximum final moisture content (%)
HAD	50	–	24	150	12.99	8.66
IRD	50	–	22	150	13.16	8.77
VD	50	0.06	21	150	12.64	8.42
MVD	35–45	−0.085	35 (min)	150	13.04	8.69
FVD	−64	0.01	20	150	12.35	8.23

Abbreviations: FVD, freeze‐vacuum drying; HAD, hot‐air drying; IRD, infrared drying; MVD, microwave‐vacuum drying; VD, vacuum drying.

### 2.3 Analysis of physicochemical properties and antioxidant activity

#### 2.3.1 Color parameters

The surface color (L*, a*, and b*) of BPA dried by five methods was detected by a colorimeter. The color parameters were expressed as L* (lightness), a* (red/green), and b* (yellow/blue).Total color difference (Δ*E*) was calculated according to the formula as described by Aghilinategh et al. (2015).

(1) $$\Delta E^{*}=\sqrt{\left(L_{0}^{*}-L^{*}\right)+\left(a_{0}^{*}-a^{*}\right)+\left(b_{0}^{*}-b^{*}\right)}$$

#### 2.3.2 Proximate compositions and free fatty acids

Moisture, protein, starch, and fat contents of dried BPA were detected according to the AOAC Official Method (2012). The free fatty acid quantification was detected by an Agilent 7890 gas chromatograph (Agilent Technologies) suggested by Wen et al. (2019).

#### 2.3.3 Bioactive components

Tetramethylpyrazine and acetoin were detected according to the method of Wen et al. (2019), using Agilent 1260 high‐performance liquid chromatography. γ‐Aminobutyric acid was analyzed using the methods of Park et al. (2017). Coixol, coixenolide, total phenolics, flavonoids, and triterpenoids were measured as suggested by Xu, Wang, et al. (2017). The contents of total phenolics and flavonoids were performed, and the results were expressed as gallic acid equivalent (mg GAE/g DW) and rutin equivalent (mg RE/g DW), respectively.

#### 2.3.4 Antioxidant activity

The ABTS^+^ and FRAP (ferric reducing antioxidant power) assays were determined using the method suggested by Szychowski et al. (2018). The DPPH radical scavenging assay was determined as previously described by Vu, Scarlett, and Vuong (2017). Briefly, dried BPA powder (1.00 g) was mixed with 80% ethanol (15 ml). The mixture was ultrasonic extracted for 30 min at room temperature, followed by centrifugation at 2500 *g* for 15 min. The supernatant was collected, and these procedures were performed in triplicate. After extraction, all the supernatants were combined and concentrated into a paste by a rotary evaporation at 45°C under reduced pressure. The pastes were reconstituted with 10 ml of methanol, and 1.0 ml of the extract was transferred to a 50‐ml volumetric flask and diluted with methanol to volume. Subsequently, the extracts were stored at −18°C until used.

### 2.4 Statistical analysis

All the determinations were performed in triplicate, and the results were shown as mean ± standard deviation (*SD*). Statistical analysis, Pearson's correlation analysis, and principal component analysis were performed by SPSS 17.0 software (SPSS Inc). Differences among the results were evaluated by Duncan's test and analysis of variance (ANOVA).

## 3 RESULTS AND DISCUSSION

### 3.1 Final moisture content and drying time

The final moisture content and drying times for different methods were displayed in Table 1, HAD, 8.66%, 24 hr; IRD, 8.77%, 22 hr; VD, 8.42%, 21 hr; MVD, 8.69%, 35 min; and FVD, 8.23%, 20 hr. MVD method had the shortest time for drying BPA samples, ~27 times lower than that of FVD. HAD method required the longest time for drying BPA samples, ~1.20 times higher than that of FVD. In MVD processing, more heat was generated within the samples under low pressure, rapidly forming a vapor pressure. And then, a vapor pressure gradient was created between the samples and surrounding, accelerated the water flow from the interior to the samples surface (Pankyamma, Mokam, Debbarma, & Rao B, 2019). Compared with HAD, a specific infrared irradiation could directly penetrate into the dried samples, resulting in a rapid temperature increase and high water evaporation in dried samples. And during HAD processing, the external heat flux transmitted from the samples surface to the interior, caused a surface hardening, and finally impeded the moisture transfer inside samples (Wang et al., 2019).

### 3.2 Chromaticity

The color parameters of BPA treated by five drying methods were revealed in Table 2, and photographs were shown in Figure S1a–e (Supplementary material). WVD (30.71) provided higher color lightness, followed by HAD (22.03), and then VD (21.93). The decreases in the L* value of IRD (18.35) indicated color darkening of dried samples. The highest and lowest a* values were found in IRD (14.74) and VD (10.19) samples, respectively. The b* value of dried BPA samples ranged from 5.48 to 10.04. The highest Δ*E* was found in IRD samples, but the lowest Δ*E* was observed in FVD samples. And there had no significant difference on the Δ*E* values for the VFD, VD, and HAD samples. Higher L* value and lower Δ*E* value were an indicator for the better color quality of dried samples (Wang, Zhang, & Mujumdar, 2014). The MVD samples had highest lightness (L*), the FVD dried samples had lowest mean changes (Δ*E*), but IRD treated samples had lowest lightness and highest mean changes. MVD processing, with the properties of less oxygen and shorter drying time, led to a less enzymatic browning reaction and Maillard reaction in dried samples (Tian, Zhao, Huang, Zeng, & Zheng, 2016). The lower ΔE value was obtained in FVD samples, mainly due to that FVD protected the integrity of cells, avoiding Maillard reaction and nonenzymatic browning reactions (Duodu, 2011). During HAD and IRD processing under high temperature and aerobic conditions, the browning was produced by enzymatic reaction and the nonenzymatic reactions, which made the sample color red and dark (Li, Zhang, & Bhandari, 2019; Wang et al., 2019). Similarly, segmented cabbages were subjected to FVD, VD, MVD, and HAD, and the order of the four methods of the ΔE values was HAD > VD > MVD > FVD (Xu et al., 2019).

**Table 2:** Physicochemical properties of BDA prepared by five drying methods

Drying methods	L*	a*	b*	ΔE	Protein (% DW)	Starch (% DW)	Fat (% DW)
HAD	22.03 ± 1.16b	11.39 ± 0.34b	5.48 ± 0.57d	12.13 ± 0.52b	10.68 ± 0.81a	26.34 ± 0.29c	6.80 ± 0.19ab
IRD	18.35 ± 0.61c	14.74 ± 0.49a	6.86 ± 0.48c	17.00 ± 0.40a	11.17 ± 0.45a	27.59 ± 0.53ab	6.79 ± 0.30ab
VD	21.93 ± 1.02b	10.19 ± 0.80c	9.70 ± 0.52ab	11.61 ± 0.33b	10.42 ± 0.54a	26.80 ± 0.40b	6.56 ± 0.19b
MVD	30.71 ± 0.74a	11.15 ± 0.71bc	10.04 ± 0.44a	11.18 ± 0.43c	11.21 ± 0.39a	27.29 ± 0.37d	7.24 ± 0.24a
FVD	21.92 ± 0.95b	10.74 ± 0.56bc	8.66 ± 0.61b	9.77 ± 0.61b	11.40 ± 0.62a	28.51 ± 0.58a	6.82 ± 0.31ab

Note

Results are expressed as mean ± standard error. Different lower‐case letters in the same column indicate significant differences at *p* < .05.

Abbreviations: FVD, freeze‐vacuum drying; HAD, hot‐air drying; IRD, infrared drying; MVD, microwave‐vacuum drying; VD, vacuum drying.

### 3.3 Proximate compositions

As displayed in Table 2, the contents of protein, starch, and fat in VD, HAD, and IRD samples were lower than that of FVD samples. The protein contents of BPA treated by five drying methods had not significant difference (*p* > .05). The highest protein content was presented in FVD sample (11.21% DW), followed by MVD, IRD, HAD, and VD. FVD samples had the highest starch content, while MVD samples had the lowest. Except VD samples, no significant difference was obtained on the fat contents of BPA samples treated by other drying methods (*p* > .05). The highest fat content was presented in MVD samples, but VD samples showed low fat content. Similarly, significant differences in proximate composition were found among different treatments, and FVD samples had higher contents of proximate composition (Gong et al., 2019; Li, Jin‐Jia, Ling, & Hao, 2019). Samples treated by FVD at low temperature condition, and the cell injury was generally negligible; thus the contents of protein, starch, and fat were higher in FVD samples (Öztürk & Gündüz, 2018). In contrast, VD, HAD, and VD drying time was so long, the cell shrinkage or expansion of the cellular structure resulted in its rupture, so there resulted in plenty of physical or chemical reactions (Li, Jin‐Jia, et al., 2019).

### 3.4 Fatty acid composition

The fatty acid compositions of dried BPA were shown in Table 3, and eighteen fatty acids were detected in dried BPA samples. The content of total fatty acids ranged from 60.76 g/100 g DW to 48.08 g/100 g DW, and the order of the five methods was FVD > VD > MVD > HAD > IRD. The contents of individual fatty acids in dried BPA samples shows markedly difference from each other (*p* < .05). Oleic acid (49.54%–53.15%) was the major fatty acid in dried BPA samples, followed by linoleic (25.20%–28.03%) and palmitic (13.77%–17.79%) acids. Similarly, Yang, Tseng, Chang, Lee, and Mau (2005) reported that oleic, linoleic, and palmitic acids were major fatty acids obtained in *monascal* adlay. There were significant differences in the contents of the saturated (SFA), monounsaturated (MUFA), and polyunsaturated (PUFA) fatty acids of dried BPA samples. The MUFA content ranged from 25.70 to 30.38 g/100 g DW, with the highest level in VD samples and lowest in IRD samples. The total PUFA content in IRD‐, HAD‐, FVD‐, VD‐, and MVD‐dried BPA increased successively, ranging from 14.00 to 16.28 g/100 g DW. FVD samples had the highest SFA content (14.47 g/100 g), but IRD resulted in the lowest one (8.37 g/100 g). The slight reduction of MUFA and PUFA in dried samples treated by HAD and IRD was due to oxidation of unsaturated fatty acids at a higher heating temperature and longer time (Suri, Singh, Kaur, Yadav, & Singh, 2019). The PUFA/SFA ratios were 1.66, 1.67, 1.23, 1.65, and 1.10 for BPA treated by HAD, IRD, VD, WVD, and FVD, respectively. The minimum recommended PUFA/SFA ratio was 0.45 for a human diet (Niu et al., 2017), indicating that BPA could provide a potential healthy diet for human.

**Table 3:** Contents of fatty acids in BDA dried by five drying methods (g/100 g DW)

Fatty acids	HAD	IRD	VD	WVD	FVD
Hexanoic	0.01 ± 0.00a	0.01 ± 0.00a	<0.01	<0.01	0.01 ± 0.01a
Octanoic (C8:0)	0.01 ± 0.01a	0.01 ± 0.00a	<0.01	<0.01	0.01 ± 0.01a
Lauric (12:0)	0.02 ± 0.00b	0.02 ± 0.02ab	0.02 ± 0.01a	0.01 ± 0.01b	0.03 ± 0.00a
Myristic (C14:0)	0.03 ± 0.01a	0.03 ± 0.02ab	0.05 ± 0.00a	0.03 ± 0.00b	0.06 ± 0.02a
Pentadecanoic (C15:0)	0.02 ± 0.01bc	0.02 ± 0.00c	0.04 ± 0.01ab	0.02 ± 0.02bc	0.05 ± 0.00a
Palmitic (C16:0)	7.17 ± 0.38c	6.62 ± 0.56d	9.86 ± 0.27b	7.82 ± 0.40c	10.81 ± 0.34a
Heptadecanoic (C17:0)	0.07 ± 0.04ab	0.07 ± 0.02b	0.10 ± 0.00a	0.07 ± 0.01b	0.12 ± 0.03a
Stearic (C18:0)	1.25 ± 0.11c	1.17 ± 0.14c	2.18 ± 0.20ab	1.39 ± 0.09c	2.41 ± 0.15a
Arachidic (C20:0)	0.25 ± 0.01b	0.29 ± 0.06b	0.48 ± 0.02a	0.33 ± 0.07b	0.56 ± 0.08a
Heneicosanoic (C21:0)	0.01 ± 0.01b	0.01 ± 0.01b	0.03 ± 0.01ab	0.02 ± 0.00b	0.04 ± 0.00a
Behenic (C22:0)	0.09 ± 0.01b	0.09 ± 0.03b	0.20 ± 0.04a	0.09 ± 0.02b	0.21 ± 0.05a
Lignoceric (C24:0)	0.06 ± 0.03c	0.05 ± 0.00d	0.16 ± 0.03b	0.06 ± 0.00d	0.17 ± 0.04a
Total SFA	8.98	8.37	13.13	9.85	14.47
Palmitoleic (C16:1)	0.12 ± 0.01a	0.12 ± 0.02a	0.10 ± 0.04a	0.13 ± 0.05a	0.11 ± 0.02a
Oleic (C18:1)	27.19 ± 0.31b	25.44 ± 0.57c	30.47 ± 0.29a	29.95 ± 0.42a	30.10 ± 0.36a
Eicosenoic (C20:1)	0.14 ± 0.05a	0.14 ± 0.02a	0.20 ± 0.04a	0.14 ± 0.03a	0.18 ± 0.05a
Total MUFA	27.44	25.70	30.77	30.23	30.38
Linoleic (C18:2)	14.39 ± 0.32c	13.47 ± 0.49d	15.46 ± 0.26b	15.70 ± 0.50b	15.31 ± 0.44b
α‐Linolenic (C18:3)	0.50 ± 0.04a	0.50 ± 0.10a	0.54 ± 0.07a	0.54 ± 0.06a	0.49 ± 0.09a
Arachidonate (C20:4)	0.03 ± 0.00c	0.03 ± 0.03c	0.08 ± 0.01b	0.03 ± 0.02c	0.10 ± 0.01a
Total PUFA	14.92	14.00	16.08	16.28	15.90
TFA	51.34	48.08	59.98	56.35	60.76
PUFA/SFA	1.66	1.67	1.23	1.65	1.10

Note

Results are expressed as mean ± standard error. Lower‐case letters in the same line indicate significant differences at *p* < .05.

Abbreviations: FVD, freeze‐vacuum drying; HAD, hot‐air drying; IRD, infrared drying; MUFA, monounsaturated fatty acid; MVD, microwave‐vacuum drying; PUFA, polyunsaturated fatty acid; SFA, saturated fatty acid; TFA, total fatty acids; VD, vacuum drying.

### 3.5 Tetramethylpyrazine and acetoin

**Figure 1:**
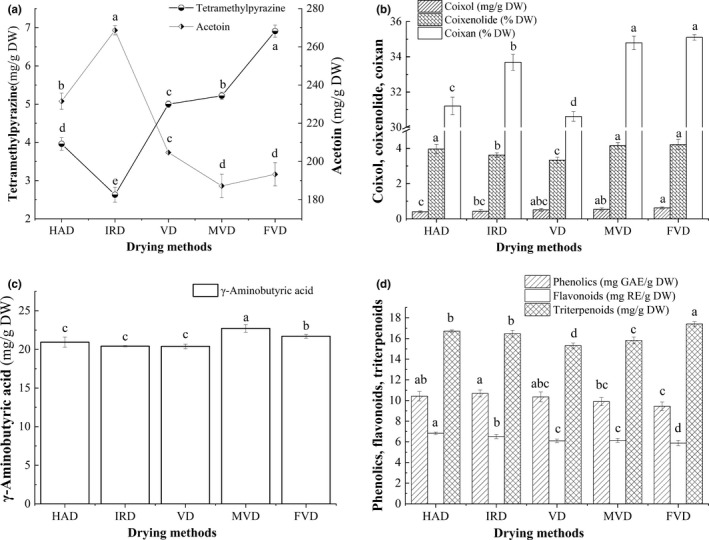
Effects of drying method on bioactive components in BPA. (a) Tetramethylpyrazine and acetoin; (b) coixol, coixenolide, and coixan; (c) γ‐aminobutyric acid; and (d) phenolics, flavonoids, and triterpenoids. HAD, hot‐air drying; IRD, infrared drying; VD, vacuum drying; MVD, microwave‐vacuum drying; and FVD, freeze‐vacuum drying

Tetramethylpyrazine naturally existed in fermented foods, such as natto, Chinese liquor, and vinegar, and had health functions especially for cardiovascular and cerebrovascular health (Li, Huang, Wang, & Qiu, 2019). Acetoin has recently been in the focus of high‐value industries because of its usability in detergents, cosmetics, dairy products, and pharmaceuticals (Lee, Jo, Song, Park, & Mun, 2019). As shown in Figure 1a, there were remarkable differences on the contents of tetramethylpyrazine and acetoin in dried BPA samples. The tetramethylpyrazine content ranged from 2.69 to 6.91 mg/g DW, with the highest content in FVD samples and lowest in IRD samples. The retention ratios of tetramethylpyrazine as compared to FVD samples were 57.31%, 38.06%, 72.36%, and 75.54% in BPA dried by HAD, IRD, VD, and WVD, respectively. In previous studies, the tetramethylpyrazine contents ranged from 0.001 to 0.131 mg/g and 0.09 to 1.42 mg/L in vinegar samples and light aroma type Chinese liquors, respectively (Chen et al., 2010; Niu et al., 2017), which were well lower than our data. The acetoin content in HAD‐, IRD‐, VD‐, MVD‐, and FVD‐processed BPA increased successively, ranging from 187.22 to 268.65 mg/g DW. Tetramethylpyrazine and acetoin were generally regarded as the important flavor compounds due to their volatile and low steam pressure (Zhu, Xu, & Fan, 2010). The lower tetramethylpyrazine content in HAD, IRD, and MVD was due to that heating and moisture evaporation both promoted the volatilization of tetramethylpyrazine during drying (Dong, Hu, Chu, Zhao, & Tan, 2017). In contrast, the higher acetoin content in HAD and IRD was detected, due to that higher heating temperature and longer time disrupted the cell wall and release acetoin from *B. subtilis* BJ3‐2.

### 3.6 Coixol, coixenolide, and coixan

Coixol, coixenolide, and coixan from adlay have attracted increasing attention due to their potential biological functions, especially antioxidant, antitumor, antidiabetic, antimelanin, and anti‐inflammatory activities (Yin, Wang, Nie, & Xie, 2018). As shown in Figure 1b, the contents of coixol, coixenolide, and coixan in BPA treated by five drying methods had significant difference (*p* < .05). The contents of coixol, coixenolide, and coixan in BPA subjected to different drying methods ranged from 0.40 to 0.62 mg/g DW, 3.62 to 4.09% DW, and 30.61 to 35.09% DW, respectively. The highest values of coixol, coixenolide, and coixan were recorded for FVD, while HAD in the lowest amount of coixol and VD in the lowest amount of coixenolide and coixan. As compared to FVD, the higher retention ratios of coixol, coixenolide, and coixan in MVD samples were 87.10%, 98.57%, and 99.11%, respectively.

### 3.7 γ‐Aminobutyric acid

γ‐Aminobutyric acid, a four‐carbon nonprotein amino acid, is a major inhibitory neurotransmitter in the sympathetic nervous system (Xie et al., 2019). As displayed in Figure 1c, the highest γ‐aminobutyric acid content was observed in MVD‐prepared samples (22.71 mg/g DW), followed by FVD (21.70 mg/g DW), but the lowest was obtained in VD samples (20.39 mg/g DW), indicating that γ‐aminobutyric acid was heat resistant component. As compared to FVD‐prepared samples, the retention ratios of γ‐aminobutyric acid in BPA samples subjected to HAD, IRD, VD, and WVD were 96.45%, 94.05%, 93.96%, and 104.66%, respectively. Chungcharoen, Prachayawarakorn, Tungtrakul, and Soponronnarit (2015) showed that the γ‐aminobutyric acid content changed insignificantly in high temperature (90–150°C).

### 3.8 Phenolics, flavonoids, and triterpenoids

Phenolics, flavonoids, and triterpenoids are an essential group of plant metabolites during development and in response to various conditions, and exhibit pharmacological effects on treating many diseases such as inflammation, cardiovascular disease, hypertension, arteriosclerosis, and cancer (Xu, Chen, et al., 2017). As noted in Figure 1d, different dehydrated treatments displayed variable effects on the contents of total phenolics, flavonoids, and triterpenoids in BPA samples. The contents of total phenolics and flavonoids of dried BPA samples were 9.45–10.69 mg GAE/g DW and 5.68–6.82 mg RE/g DW, respectively. The highest phenolic contents were presented in HAD samples, approximately 1.13 times higher than that of FVD. And the highest flavonoid content was found in IRD samples, approximately 1.60 times higher than that of FVD. It was supported by Cheng et al (2019), who also found that FVD resulted in less phenolic and flavonoid contents of green coffee beans compared with MVD and HAD, which was consistent with our results. Heating created more destruction of the tissue, which in turn led to the thermal degradation or transformation into simpler phenolic compounds, and polymerization or oxidation of phenolic and flavonoid compounds (Si et al., 2016). During the freezing step prior to FVD, the cellular structure of samples was damaged by ice crystals formation and FVD processing was treated under lower exposure to oxygen. After the end of FVD, enzymatic oxidation of phenolics was more easily to happen when exposed to air (Duodu, 2011). Additionally, lower flavonoid and phenolic contents in MVD samples were due to that heat generation from microwave radiation were rapid and intense and lead to thermal degradation of phenolics and flavonoids (Lim & Murtijaya, 2007).

The triterpenoid content in dried samples was 15.31–17.41 mg/g DW. FVD samples had the highest content of triterpenoids, but IRD resulted in the lowest one. The retention ratios of triterpenoids as compared to FVD samples were 95.88%, 94.65%, 90.80% and 87.94% in BPA dried by HAD, IRD, VD, and WVD, respectively. Li, Jin‐Jia, et al. (2019)) reported that the triterpenoids content of Eucalyptus urophylla × Eucalyptus grandis bark dried by FVD was higher than that dried by HAD. Additionally, Chen et al. (2017) reported that the triterpenoid retention in MVD, VD, atmospheric microwave drying, and HAD‐processed wax gourd peel decreased successively.

### 3.9 Antioxidant activity and Pearson's correlation analysis

**Figure 2:**
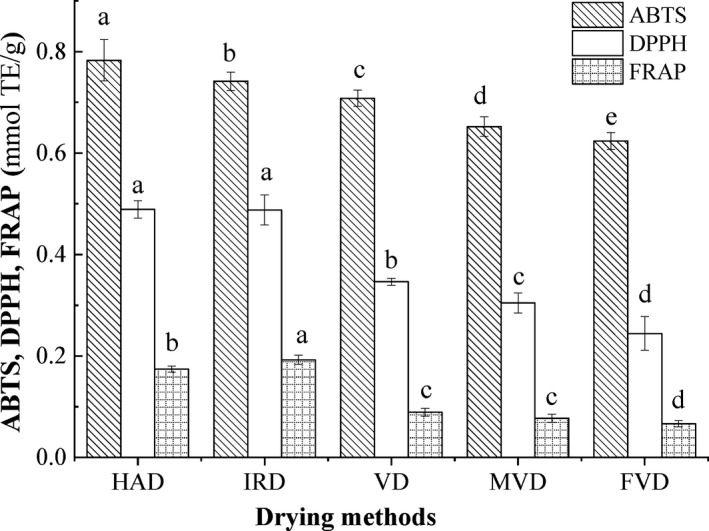
Effects of drying method on antioxidant activity in BPA. FVD, freeze‐vacuum drying; HAD, hot‐air drying; IRD, infrared drying; MVD, microwave‐vacuum drying; VD, vacuum drying

The antioxidant activity of HAD, IRD, VD, MVD, and FVD sample powders was evaluated by ABTS^+^, DPPH, and FRAP assays in BPA (Figure 2). The ABTS^+^, DPPH, and FRAP values of dried samples processed by HAD and IRD were significantly higher than that dried by VD, MVD, and FVD. The higher values of ABTS^+^ and DPPH were detected in HAD samples, the highest value of FRAP was found in IRD samples, while the lowest values of ABTS^+^, DPPH, and FRAP were observed in FVD samples. Si et al. (2016) observed that the order of ABTS^+^, DPPH, and FRAP values of raspberry powders was as follows: IRD > HAD > FVD. Rahman, Shamsudin, Ismail, Shah, and Varith (2018) revealed that new bioactive compounds and Maillard‐type antioxidants formed from their precursor at high temperature, which explained that antioxidant activities of HAD and TRD samples were higher than FVD samples. Previous studies demonstrated that the browning of fruits and vegetable was caused by Maillard reaction, and melanoidin pigment of Maillard reaction products had high antioxidant capacity (Rafiq, Singh, & Gat, 2019). Meanwhile, high temperature could promote the conversion of flavone glycoside into flavone aglycone compounds and low molecular weight phenolic compounds with stronger antioxidant activity (Ming et al., 2019). Additionally, phenolic compounds were bound to the skeleton of melanoidins of Maillard reaction products in the form of noncovalent bond and synthesized melanoidin compounds with strong antioxidant activity (Wang, Qian, & Yao, 2011). Therefore, the antioxidant activities of BPA treated by HAD and IRD were significantly higher than other drying methods.

Pearson's correlation was used to evaluate the relationship between bioactive components and antioxidant activities of dried BPA as shown in Table 4. Obviously, low or negative correlations were revealed between the antioxidant activities and the contents of tetramethylpyrazine, acetoin, coixol, coixenolide, coixan, γ‐aminobutyric acid, and triterpenoids of the dried samples, implying the antioxidant activities were insignificantly affected by those bioactive components. The positive correlation between the antioxidant activities and the contents of total phenolics and flavonoids was detected, which was better than that of DPPH situation with R^2^ value more than 0.90. The positive correlation between total phenolic and flavonoids content and the antioxidant activities was observed in previous studies. Pham, Van Nguyen, et al. (2017)), Pham, Vuong, et al. (2017) showed that the phenolic compounds and flavonoids correlated with DPPH, ABTS^+^, and FPAP (*R* ^2^ range of 0.78 and 0.99). Xu et al. (2019) observed a positive correlation between the antioxidant activities (DPPH and ORAC) and the contents of total phenolics and flavonoids. This was also supported by the study of Rafiq et al. (2019), who have indicated that the flavonoid with hydroxyl groups was well‐displayed radical scavenging activity.

**Table 4:** Pearson's correlation coefficients between the bioactive components and antioxidant activities of dried BPA

Antioxidant activity	MTP	Acetoin	Coixol	Coixenolide	Coixan	GABA	Phenolics	Flavonoids	Triterpenoids
ABTS	−.449	.493	−.449	.493	−.784	−.676	.884*	.881*	−.125
DPPH	−.689	.683	−.689	.683	−.504	−.617	.906*	.906*	−.045
FRAP	−.665	.835	−.665	.835	−.191	−.594	.868*	.859*	.132

* Represents the significant correlation (*p* < .05).

### 3.10 Principal component analysis

**Figure 3:**
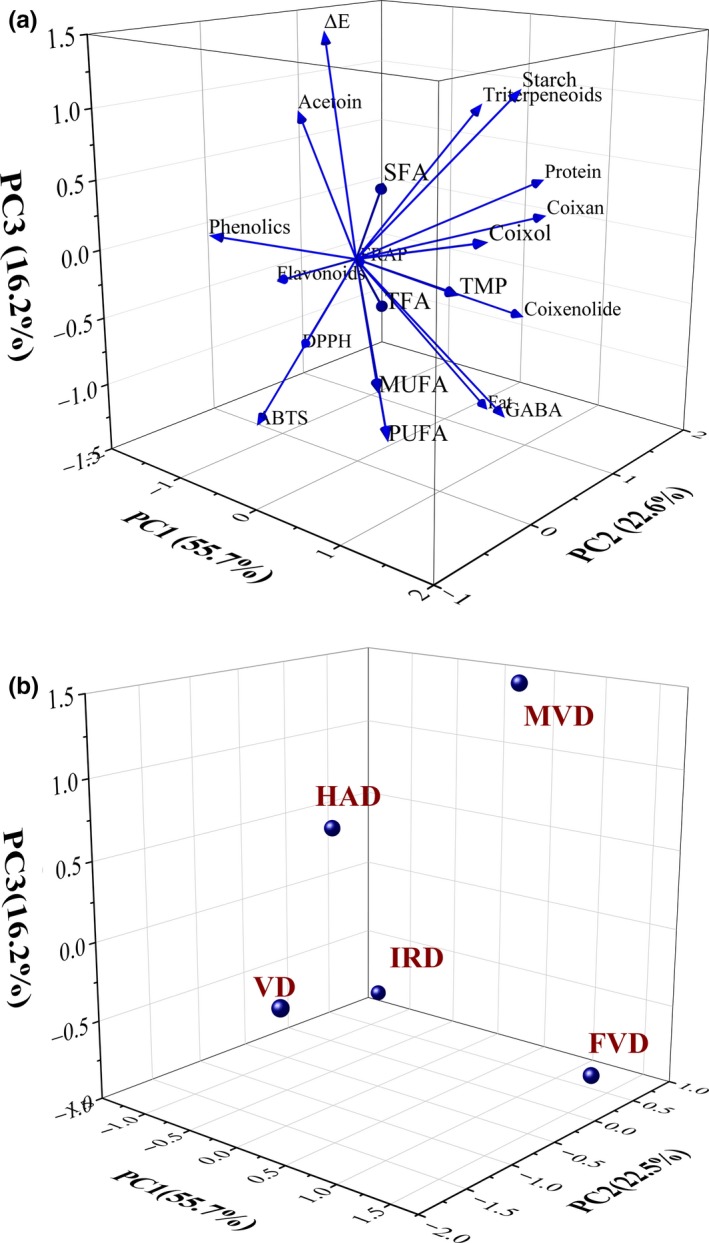
Principal component analysis loading plot (a) and score plot (b) describing relationship among different properties of BPA treated by five drying methods. FVD, freeze‐vacuum drying; HAD, hot‐air drying; IRD, infrared drying; MVD, microwave‐vacuum drying; VD, vacuum drying

Principal component analysis (PCA) was used to investigate the comprehensive quality of dried BPA samples processed by the different drying methods. Figure 3a, b showed the loading plots and scores defined by the three PCs obtained from the PCA, and the three principal components explained 94.48% of total variance. The three principal components accounted for 55.69%, 22.55%, and 16.24% of the total variance, respectively. Tetramethylpyrazine, coixol, total free fatty acids, and phenolics contributed to a large extent to PC1. Protein and coixan were closely related to PC2, and fat and ΔE were closely related to PC3 as displayed in Table S1 (Supplementary material). As shown in Table S2 (Supplementary material), the result showed that the samples of the top three categories of comprehensive quality were BPA by FVD, MVD, and VD, respectively. FVD samples displayed the best comprehensive quality, but high cost of FVD might limit its application. Therefore, MVD could be an alternative preservation method when considering the economy and drying time.

## 4 CONCLUSION

The nutritional and bioactive components, and antioxidant activity of BPA processed by five drying methods were evaluated. The higher contents of tetramethylpyrazine, coixol, coixenolide, coixan, and triterpenoids were detected in FVD samples. The higher contents of total phenolics and flavonoids, and antioxidant activity were exhibited in HAD and IRD samples. The shorter time, higher γ‐aminobutyric acid content, and higher retention rate of tetramethylpyrazine, coixol, coixenolide, and coixan were obtained in MVD samples. Through principal component analysis, BPA subjected to FVD, MVD, and VD had higher comprehensive quality. MVD was a promising technology for preserving BPA for short drying time and high bioactive components. For further work, it is certainly worth examined that the change rules and kinetic models of bioactive components loss in BPA.

## CONFLICT OF INTEREST

The authors declare no financial or commercial conflict of interest.

## ETHICAL APPROVAL

This study does not involve any human or animal testing.

## INFORMED CONSENT

This study does not require informed consent because it does not use humans as research subjects.

## Supporting information

Figure S1

Tables S1‐S2

## References

[fsn31508-bib-0001] Aghilinategh, N. , Rafiee, S. , Gholikhani, A. , Hosseinpur, S. , Omid, M. , Mohtasebi, S. S. , … Maleki, N. (2015). A comparative study of dried apple using hot air, intermittent and continuous microwave: Evaluation of kinetic parameters and physicochemical quality attributes. Food Science & Nutrition, 3(6), 519–526. 10.1002/fsn3.241 26788293 PMC4708644

[fsn31508-bib-0002] Chen, J. , Chen, Q. , Guo, Q. , Ruan, S. , Ruan, H. , He, G. , … Gu, Q. (2010). Simultaneous determination of acetoin and tetramethylpyrazine in traditional vinegars by HPLC method. Food Chemistry, 122(4), 1247–1252. 10.1016/j.foodchem.2010.03.072

[fsn31508-bib-0003] Chen, Y. , Li, J. , Fu, F. , Wang, N. , Wang, D. , Qin, D. , … Fan, H. (2017). Effects of different drying methods on the content of active components of wax gourd peel. Food and Fermentation Industries, 43(02), 129–133. 10.13995/j.cnki.11-1802/ts.201702022

[fsn31508-bib-0004] Cheng, K. , Dong, W. , Long, Y. , Zhao, J. , Hu, R. , Zhang, Y. , … Zhu, K. (2019). Evaluation of the impact of different drying methods on the phenolic compounds, antioxidant activity, and in vitro digestion of green coffee beans. Food Science & Nutrition, 7(3), 1084–1095. 10.1002/fsn3.948 30918651 PMC6418437

[fsn31508-bib-0005] Chungcharoen, T. , Prachayawarakorn, S. , Tungtrakul, P. , & Soponronnarit, S. (2015). Effects of germination time and drying temperature on drying characteristics and quality of germinated paddy. Food and Bioproducts Processing, 94, 707–716. 10.1016/j.fbp.2014.09.013

[fsn31508-bib-0006] Ding, Y. , Pu, L. , & Kan, J. (2017). Hypolipidemic effects of lipid‐lowering granulated tea preparation from Monascus‐fermented grains (adlay and barley bran) mixed with lotus leaves on Sprague‐Dawley rats fed a high‐fat diet. Journal of Functional Foods, 32, 80–89. 10.1016/j.jff.2017.02.025

[fsn31508-bib-0007] Dong, W. , Hu, R. , Chu, Z. , Zhao, J. , & Tan, L. (2017). Effect of different drying techniques on bioactive components, fatty acid composition, and volatile profile of robusta coffee beans. Food Chemistry, 234, 121–130. 10.1016/j.foodchem.2017.04.156 28551215

[fsn31508-bib-0009] Duodu, K. G. (2011). Effects of processing on antioxidant phenolics of cereal and legume grains (vol. 1089). In Advances in Cereal Science: Implications to Food Processing and Health Promotion, pp. 31–54. 10.1021/bk-2011-1089.ch003

[fsn31508-bib-0010] Gong, X. , Huang, X. , Yang, T. , Wen, J. , Zhou, W. , & Li, J. (2019). Effect of drying methods on physicochemical properties and antioxidant activities of okra pods. Journal of Food Processing and Preservation, 10.1111/jfpp.14277

[fsn31508-bib-0011] Huang, Q. , Xu, M. , Zhang, H. , He, D. , Kong, Y. , Chen, L. , … Song, H. (2019). Transcriptome and proteome analyses of the molecular mechanisms associated with coix seed nutritional quality in the process of breeding. Food Chemistry, 272, 549–558. 10.1016/j.foodchem.2018.07.116 30309580

[fsn31508-bib-0012] Lee, C. , Jo, C. Y. , Song, Y. J. , Park, H. , & Mun, S. (2019). Optimal design of a simulated‐moving‐bed chromatographic process for high‐purity separation of acetoin from 2,3‐butanediol in a continuous mode. Journal of Chromatography A, 1607 10.1016/j.chroma.2019.460394. in press. 31400841

[fsn31508-bib-0013] Li, D. , Huang, W. , Wang, C. , & Qiu, S. (2019). The complete genome sequence of the thermophilic bacterium *Laceyella sacchari* FBKL4.010 reveals the basis for tetramethylpyrazine biosynthesis in Moutai‐flavor Daqu. MicrobiologyOpen. 10.1002/mbo3.922. in press. PMC6925174 31482696

[fsn31508-bib-0014] Li, J. , Jin‐Jia, G. , Ling, M. , & Hao, C. (2019). Effects of drying methods on triterpenoids content and molluscicidal activity of *Eucalyptus urophylla* × *Eucalyptus grandis* bark extracts. Natural Product Research and Development, 31(09), 1641‐1646. http://kns.cnki.net/kcms/detail/51.1335.q.20190706.1532.006.html

[fsn31508-bib-0015] Li, L. , Zhang, M. , & Bhandari, B. (2019). Influence of drying methods on some physicochemical, functional and pasting properties of Chinese yam flour. LWT, 111, 182–189. 10.1016/j.lwt.2019.05.034

[fsn31508-bib-0016] Lim, Y. Y. , & Murtijaya, J. (2007). Antioxidant properties of *Phyllanthus amarus* extracts as affected by different drying methods. LWT‐Food Science and Technology, 40(9), 1664–1669. 10.1016/j.lwt.2006.12.013

[fsn31508-bib-0017] Lin, L. , Hsiao, E. S. L. , Tseng, H. , Chung, M. , Chua, A. C. N. , Kuo, M. , … Tzen, J. T. C. (2009). Molecular cloning, mass spectrometric identification, and nutritional evaluation of 10 coixins in Adlay (*Coix lachryma‐jobi* L.). Journal of Agricultural and Food Chemistry, 57(22), 10916–10921. 10.1021/jf903025n 19919123

[fsn31508-bib-0018] Ming, Z. , Mingsheng, X. , Jinyin, C. , Yonggen, S. , Meixiang, Y. , Xiaojuan, Z. , Fengni, Z. (2019). Investigation on drying kinetics of ‘Xiushui Huahong’ sweet orange peel during hot air drying process and its analysis on the quality characteristics. Food Science. 10.7506/spkx1002-6630-20190710-132

[fsn31508-bib-0019] Niu, Y. , Yao, Z. , Xiao, Q. , Xiao, Z. , Ma, N. , & Zhu, J. (2017). Characterization of the key aroma compounds in different light aroma type Chinese liquors by GC‐olfactometry, GC‐FPD, quantitative measurements, and aroma recombination. Food Chemistry, 233, 204–215. 10.1016/j.foodchem.2017.04.103 28530568

[fsn31508-bib-0020] Öztürk, F. , & Gündüz, H. (2018). The effect of different drying methods on chemical composition, fatty acid, and amino acid profiles of sea cucumber (*Holothuria tubulosa* Gmelin, 1791). Journal of Food Processing and Preservation, 42(9), e13723 10.1111/jfpp.13723

[fsn31508-bib-0021] Pankyamma, V. , Mokam, S. Y. , Debbarma, J. , & Rao B, M. (2019). Effects of microwave vacuum drying and conventional drying methods on the physicochemical and microstructural properties of squid shreds. Journal of the Science of Food and Agriculture, 99(13), 5778–5783. 10.1002/jsfa.9846 31162679

[fsn31508-bib-0022] Park, N. , Lee, T. , Nguyen, T. T. H. , An, E. , Kim, N. M. , You, Y. , … Kim, D. (2017). The effect of fermented buckwheat on producinglcarnitine‐ and γ‐aminobutyric acid (GABA)‐enriched designer eggs. Journal of the Science of Food and Agriculture, 97(9), 2891–2897. 10.1002/jsfa.8123 27790703

[fsn31508-bib-0023] Pattanagul, P. , Pinthong, R. , Phianmongkhol, A. , & Tharatha, S. (2008). Mevinolin, citrinin and pigments of adlay angkak fermented by *Monascus* sp. International Journal of Food Microbiology, 126(1), 20–23. 10.1016/j.ijfoodmicro.2008.04.019 18538878

[fsn31508-bib-0024] Pham, H. N. T. , Nguyen, V. , Vuong, Q. , Bowyer, M.C. , Scarlett, C.J. (2017). Bioactive Compound Yield and Antioxidant Capacity of Helicteres hirsuta Lour. Stem as Affected by Various Solvents and Drying Methods. Journal of Food Processing and Preservation, 41(5), e13199 10.1111/jfpp.13199

[fsn31508-bib-0025] Pham, H. N. T. , Vuong, Q. V. , Bowyer, M. C. , & Scarlett, C. J. (2017). Effect of extraction solvents and thermal drying methods on bioactive compounds and antioxidant properties of *Catharanthus roseus* (L.) G. Don (Patricia White cultivar). Journal of Food Processing and Preservation, 41(5), e13199 10.1111/jfpp.13199

[fsn31508-bib-0027] Rafiq, S. , Singh, B. , & Gat, Y. (2019). Effect of different drying techniques on chemical composition, color and antioxidant properties of kinnow (*Citrus reticulata*) peel. Journal of Food Science and Technology, 56(5), 2458–2466. 10.1007/s13197-019-03722-9 31168128 PMC6525721

[fsn31508-bib-0028] Rahman, N. F. A. , Shamsudin, R. , Ismail, A. , Shah, N. N. A. K. , & Varith, J. (2018). Effects of drying methods on total phenolic contents and antioxidant capacity of the pomelo (*Citrus grandis* (L.) Osbeck) peels. Innovative Food Science & Emerging Technologies, 50, 217–225. 10.1016/j.ifset.2018.01.009

[fsn31508-bib-0029] Si, X. , Chen, Q. , Bi, J. , Wu, X. , Yi, J. , Zhou, L. , … Li, Z. (2016). Comparison of different drying methods on the physical properties, bioactive compounds and antioxidant activity of raspberry powders. Journal of the Science of Food and Agriculture, 96(6), 2055–2062. 10.1002/jsfa.7317 26108354

[fsn31508-bib-0030] Sui, W. , Mu, T. , Sun, H. , & Yang, H. (2019). Effects of different drying methods on nutritional composition, physicochemical and functional properties of sweet potato leaves. Journal of Food Processing and Preservation, 43(3), e13884 10.1111/jfpp.13884

[fsn31508-bib-0031] Suri, K. , Singh, B. , Kaur, A. , Yadav, M. P. , & Singh, N. (2019). Impact of infrared and dry air roasting on the oxidative stability, fatty acid composition, Maillard reaction products and other chemical properties of black cumin (*Nigella sativa* L.) seed oil. Food Chemistry, 295, 537–547. 10.1016/j.foodchem.2019.05.140 31174793

[fsn31508-bib-0032] Szychowski, P. J. , Lech, K. , Sendra‐Nadal, E. , Hernández, F. , Figiel, A. , Wojdyło, A. , … Carbonell‐Barrachina, Á. A. (2018). Kinetics, biocompounds, antioxidant activity, and sensory attributes of quinces as affected by drying method. Food Chemistry, 255, 157–164. 10.1016/j.foodchem.2018.02.075 29571462

[fsn31508-bib-0033] Tian, Y. , Zhao, Y. , Huang, J. , Zeng, H. , & Zheng, B. (2016). Effects of different drying methods on the product quality and volatile compounds of whole shiitake mushrooms. Food Chemistry, 197, 714–722. 10.1016/j.foodchem.2015.11.029 26617008

[fsn31508-bib-0034] Ting, Y. , Hu, Y. T. , Hu, J. Y. , Chang, W. C. , Huang, Q. , & Hsieh, S. C. (2019). Nanoemulsified adlay bran oil reduces tyrosinase activity and melanin synthesis in B16F10 cells and zebrafish. Food Science & Nutrition, 7(10), 3216–3223. 10.1002/fsn3.1176 31660135 PMC6804758

[fsn31508-bib-0035] Vu, H. T. , Scarlett, C. J. , & Vuong, Q. V. (2017). Effects of drying conditions on physicochemical and antioxidant properties of banana (*Musa cavendish*) peels. Drying Technology, 35(9), 1141–1151. 10.1080/07373937.2016.1233884

[fsn31508-bib-0036] Wang, C. , Lin, H. , & Wu, S. (2011). Influence of dietary supplementation with Bacillus‐fermented adlay on lipid metabolism, antioxidant status and intestinal microflora in hamsters. Journal of the Science of Food and Agriculture, 91 (12): 2271‐2276. 10.1002/jsfa.4450 21618546

[fsn31508-bib-0037] Wang, C. , Wu, S. , & Shyu, Y. (2014). Antioxidant properties of certain cereals as affected by food‐grade bacteria fermentation. Journal of Bioscience and Bioengineering, 117(4), 449–456. 10.1016/j.jbiosc.2013.10.002 24216458

[fsn31508-bib-0038] Wang, H. , Qian, H. , & Yao, W. (2011). Melanoidins produced by the Maillard reaction: Structure and biological activity. Food Chemistry, 128(3), 573–584. 10.1016/j.foodchem.2011.03.075

[fsn31508-bib-0039] Wang, H. , Zhang, M. , & Mujumdar, A. S. (2014). Comparison of three new drying methods for drying characteristics and quality of shiitake mushroom (*Lentinus edodes*). Drying Technology, 32(15), 1791–1802. 10.1080/07373937.2014.947426

[fsn31508-bib-0040] Wang, Q. , Li, S. , Han, X. , Ni, Y. , Zhao, D. , & Hao, J. (2019). Quality evaluation and drying kinetics of shitake mushrooms dried by hot air, infrared and intermittent microwave–assisted drying methods. LWT, 107, 236–242. 10.1016/j.lwt.2019.03.020

[fsn31508-bib-0041] Wen, A. , Xie, C. , Mazhar, M. , Zhu, Y. , Zeng, H. , Qin, L. , … Zhu, Y. (2019). Comparative evaluation of drying methods on kinetics, biocompounds and antioxidant activity of *Bacillus subtilis*‐fermented dehulled adlay. Drying Technology, 1–11, 10.1080/07373937.2019.1648292

[fsn31508-bib-0042] Xie, W. , Ashraf, U. , Zhong, D. , Lin, R. , Xian, P. , Zhao, T. , … Mo, Z. (2019). Application of γ‐aminobutyric acid (GABA) and nitrogen regulates aroma biochemistry in fragrant rice. Food Science & Nutrition, 7(11), 3784–3796. 10.1002/fsn3.1240 31763028 PMC6848825

[fsn31508-bib-0043] Xu, L. , Chen, L. , Ali, B. , Yang, N. A. , Chen, Y. , Wu, F. , … Xu, X. (2017). Impact of germination on nutritional and physicochemical properties of adlay seed (*Coixlachryma‐jobi* L.). Food Chemistry, 229, 312–318. 10.1016/j.foodchem.2017.02.096 28372179

[fsn31508-bib-0044] Xu, L. , Wang, P. , Ali, B. , Yang, N. , Chen, Y. , Wu, F. , … Xu, X. (2017). Changes of the phenolic compounds and antioxidant activities in germinated adlay seeds. Journal of the Science of Food and Agriculture, 97(12), 4227–4234. 10.1002/jsfa.8298 28251647

[fsn31508-bib-0045] Xu, Y. , Xiao, Y. , Lagnika, C. , Li, D. , Liu, C. , Jiang, N. , … Zhang, M. (2019). A comparative evaluation of nutritional properties, antioxidant capacity and physical characteristics of cabbage (*Brassica oleracea* var. *capitate* Var L.) subjected to different drying methods. Food Chemistry, 309, 124935 10.1016/j.foodchem.2019.06.002 31732250

[fsn31508-bib-0046] Yang, J. , Tseng, Y. , Chang, H. , Lee, Y. , & Mau, J. (2005). Storage stability of monascal adlay. Food Chemistry, 90(1–2), 303–309. 10.1016/j.foodchem.2004.03.053

[fsn31508-bib-0047] Yang, J. , Tseng, Y. , Lee, Y. , & Mau, J. (2006). Antioxidant properties of methanolic extracts from monascal rice. LWT ‐ Food Science and Technology, 39(7), 740–747. 10.1016/j.lwt.2005.06.002

[fsn31508-bib-0048] Yin, H. , Wang, S. , Nie, S. , & Xie, M. (2018). Coix polysaccharides: Gut microbiota regulation and immunomodulatory. Bioactive Carbohydrates and Dietary Fibre, 16, 53–61. 10.1016/j.bcdf.2018.04.002

[fsn31508-bib-0049] Zhu, B. , Xu, Y. , & Fan, W. (2010). High‐yield fermentative preparation of tetramethylpyrazine by Bacillus sp. using an endogenous precursor approach. Journal of Industrial Microbiology & Biotechnology, 37(2), 179–186. 10.1007/s10295-009-0661-5 19904566

[fsn31508-bib-0050] Zhu, F. (2017). Coix: Chemical composition and health effects. Trends in Food Science & Technology, 61, 160–175. 10.1016/j.tifs.2016.12.003

