# Supplementary figures and images for: Comprehensive evaluation of physicochemical properties and antioxidant activity of B. subtilis‐fermented polished adlay subjected to different drying methods
Source: Food Sci Nutr. 2020 Mar 14;8(4):2124–33. doi: 10.1002/fsn3.1508 (PMC7174208; doi:10.1002/fsn3.1508)

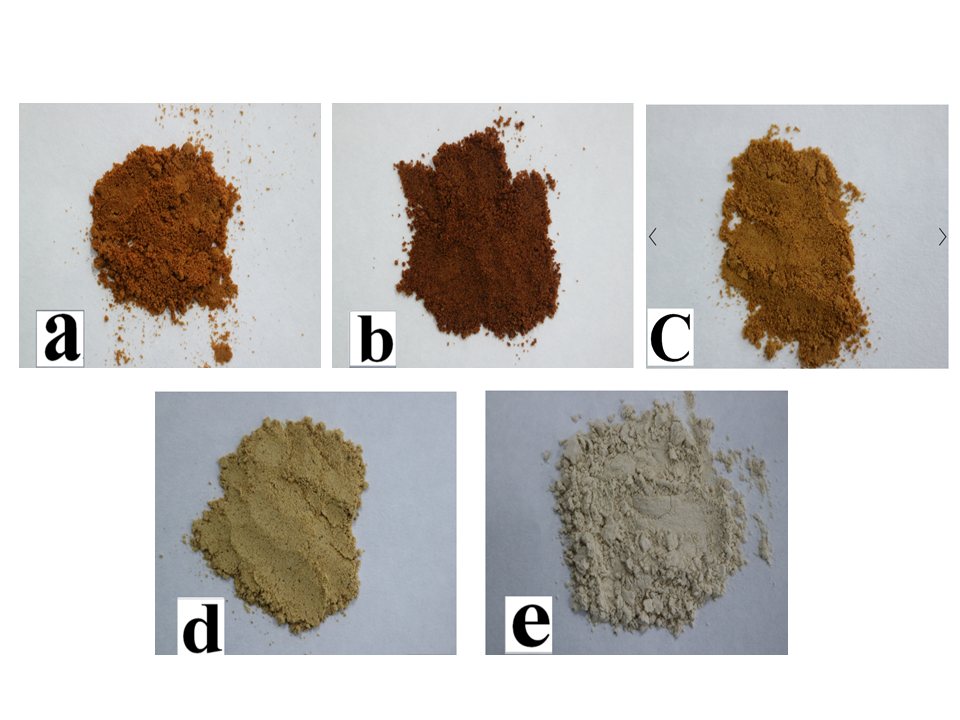

Supplement: Supplementary file 1 — Figure S1 [file FSN3-8-2124-s001.tif]
