# Supplementary material for: Comprehensive evaluation of physicochemical properties and antioxidant activity of B. subtilis‐fermented polished adlay subjected to different drying methods
Source: Food Sci Nutr. 2020 Mar 14;8(4):2124–33. doi: 10.1002/fsn3.1508 (PMC7174208; doi:10.1002/fsn3.1508)
Supplement: Supplementary file 2 — Tables S1‐S2 [file FSN3-8-2124-s002.doc]

**Supplementary materials**

**Table 1** Loading matrix of the principle component analysis

| [Quality](javascript:;) [index](javascript:;)es | PC1 | PC2 | PC3 |
| --- | --- | --- | --- |
| **Δ**E | -0.659 | 0.228 | -0.701 |
| Protein | 0.378 | 0.903 | -0.153 |
| Starch | 0.604 | 0.552 | -0.533 |
| Fat | 0.218 | 0.670 | 0.692 |
| SFA | 0.840 | -0.382 | -0.380 |
| MUFS | 0.867 | -0.423 | 0.246 |
| PUFA | 0.829 | -0.351 | 0.427 |
| TFA | 0.907 | -0.419 | -0.014 |
| TMP | 0.978 | -0.077 | 0.008 |
| Acetoin | -0.885 | 0.205 | -0.417 |
| Coixol | 0.972 | 0.089 | -0.128 |
| Coixenolide | 0.484 | 0.702 | 0.310 |
| Coixan | 0.485 | 0.839 | -0.039 |
| GABA | 0.608 | 0.494 | 0.621 |
| Phenolics | -0.952 | -0.246 | -0.042 |
| Flavonoids | -0.893 | 0.073 | 0.180 |
| Triterpenoids | 0.178 | 0.624 | -0.452 |
| ABTS | -0.739 | -0.137 | 0.651 |
| DPPH | -0.863 | 0.193 | 0.461 |
| FRAP | -0.793 | 0.508 | 0.188 |

Note: saturated fatty acids, SFA; monounsaturated fatty acids, MUFA; and polyunsaturated fatty acids, PUFA; total fatty acids, TFA; tetramethylpyrazine, TMP; γ-aminobutyric acid, GABA.

**Table 2** The composite scores of principal components after standardization for BPA dried by different methods

| Drying methods | Z_PC1_ | Z_PC2_ | Z_PC3_ | Z | Ranking |
| --- | --- | --- | --- | --- | --- |
| HAD | -0.82 | -0.15 | 0.50 | -0.41 | 4 |
| IRD | -1.14 | 0.74 | -0.85 | -0.6 | 5 |
| VD | 0.14 | -1.68 | -0.20 | -0.33 | 3 |
| MVD | 0.48 | 0.58 | 1.46 | 0.64 | 2 |
| FVD | 1.33 | 0.51 | -0.92 | 0.71 | 1 |

Note: HAD, hot air drying; IRD, infrared drying; VD, vacuum drying; MVD, microwave-vacuum drying; FVD, freeze-vacuum drying.

**Figure 1** The photographs of BPA treated by different drying methods (a, HAD; b, IRD; c, VD; d, MVD; e, FVD)

Note: HAD, hot air drying; IRD, infrared drying; VD, vacuum drying; MVD, microwave-vacuum drying; FVD, freeze-vacuum drying.
